# Regulation of Murine Natural Killer Cell Development

**DOI:** 10.3389/fimmu.2017.00130

**Published:** 2017-02-15

**Authors:** Wilford Goh, Nicholas D. Huntington

**Affiliations:** ^1^The Walter and Eliza Hall Institute of Medical Research, Parkville, VIC, Australia; ^2^Department of Medical Biology, University of Melbourne, Melbourne, VIC, Australia

**Keywords:** NK cell, transcription factors, ontogeny, maturation, homeostasis, IL-15

## Abstract

Natural killer (NK) cells are effector lymphocytes of the innate immune system that are known for their ability to kill transformed and virus-infected cells. NK cells originate from hematopoietic stem cells in the bone marrow, and studies on mouse models have revealed that NK cell development is a complex, yet tightly regulated process, which is dependent on both intrinsic and extrinsic factors. The development of NK cells can be broadly categorized into two phases: lineage commitment and maturation. Efforts to better define the developmental framework of NK cells have led to the identification of several murine NK progenitor populations and mature NK cell subsets, each defined by a varied set of cell surface markers. Nevertheless, the relationship between some of these NK cell subsets remains to be determined. The classical approach to studying both NK cell development and function is to identify the transcription factors involved and elucidate the mechanistic action of each transcription factor. In this regard, recent studies have provided further insight into the mechanisms by which transcription factors, such as ID2, FOXO1, Kruppel-like factor 2, and GATA-binding protein 3 regulate various aspects of NK cell biology. It is also becoming evident that the biology of NK cells is not only transcriptionally regulated but also determined by epigenetic alterations and posttranscriptional regulation of gene expression by microRNAs. This review summarizes recent progress made in NK development, focusing primarily on transcriptional regulators and their mechanistic actions.

## Introduction to Natural Killer (NK) Cells

Natural killer cells in mice were first described in 1975 (1–3), following further investigation into splenocytes that were able to kill tumor and virus-infected cells without prior sensitization (4–6). NK cells exert their cytotoxic effect on target cells by inducing apoptosis. Upon formation of an immunological synapse with the target cell, NK cells become activated and release cytolytic granules containing perforin and granzymes (7–9). Perforin forms pores in the membrane of target cells, thereby allowing granzymes to enter the cell, activate caspases, and initiate apoptosis (8). In a similar process known as antibody-dependent cell cytotoxicity, NK cells are able to release cytolytic granules and initiate apoptosis in opsonized cells, following recognition of the opsonized cells *via* low-affinity Fc receptors (CD16) expressed on the surface of NK cells (10). NK cells can also initiate apoptosis in target cells through the respective engagement of Fas ligands and tumor necrosis factor-related apoptosis-inducing ligand (TRAIL) on their cell surface with Fas and TRAIL receptors on the target cells (11, 12). In addition to inducing apoptosis, NK cells can indirectly mediate the clearance of target cells by producing pro-inflammatory cytokines [e.g., interferon-gamma (IFN-γ)], which boost the innate response and recruit adaptive immune responses (13–15).

The surface markers that are commonly used to identify murine NK cells by flow cytometry vary depending on the mouse strain. C57B/6 and SJL mice express the surface markers NK1.1, NKp46, and CD49b, but not CD3, which is a surface marker of T cells. CD3 is used to exclude contaminating T cell subsets, such as natural killer T cells and NK-like T cells, that, respectively, express NK1.1 and NKp46 (16). As for other mouse strains, such as BALB/c, NK cells are identified with only CD49b and NKp46 as these strains possess allelic variants of NK1.1 that cannot be detected with the widely used PK136 antibody (16, 17).

## Murine NK Cell Development

Murine NK cells can be found in all lymphoid organs and many non-lymphoid tissues, such as salivary glands, liver, and kidney. The more recent discovery of related innate lymphoid cells (ILCs) places NK cells within this family, specifically in the IL-15 dependent, IFN-γ producing group 1 ILCs. ILCs are lymphoid cells that lack rearranged antigen receptors and are dependent on the transcription factors inhibitor of DNA-binding 2 (ID2) and nuclear factor, interleukin 3 regulated (NFIL3) for their development. While NK cells are phenotypically heterogeneous and previously categorized based on their tissue of origin or location (bone marrow, thymus, fetal liver, adult liver), we appreciate that some of this heterogeneity stems from NK cells (Eomes^+^) and other ILC1s (Eomes^−^) being viewed as the same cell type. As much of our current understanding of murine NK cell development is built upon studies on bone marrow-derived NK cells [referred to here as conventional NK (cNK) cells], which represent the majority of NK cells within the body, this review will focus primarily on progress made in our understanding of cNK development.

### cNK Development in the Bone Marrow—Lineage Commitment

Conventional NK cells develop from HSCs in the bone marrow, through a sequential order of intermediate progenitors. The first progenitor to arise from HSCs is the lymphoid-primed multipotent progenitor, which then gives rise to the common lymphoid progenitor (CLP) (18). The earliest NK lineage committed progenitor to arise from CLPs is known as pre-pro NK (19), which was subdivided into pre-pro A and pre-pro B (19, 20). Differing only in c-kit (CD117) expression, the relationship between pre-pro A and B remains unclear and requires further investigation. Pre-pro NK cells then differentiate into the NK progenitor (NKP) (19, 21). NKPs give rise to immature NK (iNK) cells that either undergo further development within the bone marrow (22) or enter the periphery and develop into mature NK cells (23, 24).

As the early stages of murine NK development are still poorly defined, the developmental pathway outlined above is by no means the definitive model. Heterogeneity within existing progenitor populations, along with the discovery of new distinguishing cell markers, have led to the identification of new sub-populations and, therefore, refinements to the developmental pathway of NK cells. For instance, the common innate lymphoid progenitor (CILP) was found to possess the capacity to give rise to all lineages within the ILC family, of which NK cells are the founding member, but not B and T cells, thus making it an earlier progenitor than the pre-pro NK (25). As the CILP is expresses α_4_β_7_, it is also alternatively referred to as the α-lymphoid precursor (αLP) (25). However, it has been postulated that there could be an even earlier progenitor population, as there were only about 50 CILPs per mice, and only 2.5% of CILPs efficiently developed into all ILC lineages (25).

The early innate lymphoid progenitor (EILP) is proposed to be the earliest known progenitor for ILCs and was identified using a TCF1 (T-cell factor 1, encoded by *Tcf7*) transgenic mouse strain that expresses a green fluorescent protein reporter (26). Like CILPs, EILPs give rise to all ILC lineages both *in vivo* and *in vitro*, albeit more efficiently (26). Nonetheless, EILPs have not been shown to differentiate into CILPs and hence, the relationship between these two progenitors remains unresolved. As most of the surface markers used to identify the EILP and CILP were different, a detailed comparison of the surface marker phenotype between the two progenitors may also provide further insight.

The discovery of pre–pro NKPs was the outcome of efforts to better understand why only 8–40% of NKPs had solely NK cell potential (19). Similar studies resulted in the identification of a pre-NKP population that preceded a “streamlined” population of NKPs known as refined-NKP (rNKP) (27). Even though many parallels have been drawn between the pre-NKP and pre-pro NK, likewise with NKP and rNKP, it remains to be determined if these populations are exactly identical (20). A summary of the surface markers that are expressed on the various progenitors are provided in Table 1.

**Table 1:** **Surface markers expressed by different natural killer (NK) cell populations reported in the literature**.

Surface markers	Common lymphoid progenitor	Early innate lymphoid progenitor	Common innate lymphoid progenitor (CILP)/αLP	Pre–pro A NK	Pre–pro B NK	NK progenitor (NKP)	pre-NKP	refined-NKP (rNKP)	Imm NK	M1 NK	M2 NK
NK1.1	−	−	−	−	−	−	−	−	+	+	+
CD11b (MAC-1)	−	−	−	−	−	−	−	−	−	+	+
CD127 (IL-7Rα)	hi	lo	hi	hi	hi	hi	hi	int	int	lo	lo
CD117 (c-kit)	int	lo	int	int	lo	lo	int	lo	int	lo	lo
Sca-1	int	−	int	+	+	+	+	+	−	−	−
CD49b	−	−	−	−	−	−	−	−	−	+	+
CD27	+	ND	+	+	+	+	+	+	+	+	−
CD244 (2B4)	+	ND	+	+	+	+	+	+	+	+	+
CD25 (IL-2Rα)	−	−	−	ND	ND	−	−	+	−	−	−
CD122 (IL-2Rβ)	−	−	−	−	−	+	−	+	+	+	+
CD132 (IL-2Rγ_c_)	+	+	+	+	+	+	+	+	+	+	+
CD314 (NKG2D)	−	−	−	+	+	+	+	+	+	+	+
CD226 (DNAM1)	−	ND	int	ND	ND	+	ND	ND	hi	int	lo
CD279 (PD1)	−	ND	−/+	ND	ND	hi	ND	ND	−	−	−
CD43 (Leukosialin)	−	−	−	−	−	−	−	−	lo	int	hi
CD335 (NKp46)	−	−	−	−	−	−	−	−	+	+	+
CD253 (tumor necrosis factor-related apoptosis-inducing ligand)	−	ND	ND	ND	ND	ND	ND	ND	int	lo	lo
KLRG1	−	ND	ND	−	−	−	ND	ND	−	−	+
α_4_β_7_ (LPAM)	+	+	+	ND	ND	−	ND	ND	−	−	−
Ly49s	−	−	−	−	−	−	−	−	−/+	−/+	−/+
CD94-NKG2	−	ND	ND	−	−	−	−	−	+	+	+
CD62L (L-selectin)	−	ND	ND	−	−	−	ND	ND	−/+	+	+
CD146 (MCAM)	−	ND	ND	−	−	−	ND	ND	lo	int	hi
CXCR3	−	ND	ND	−	−	+	ND	ND	hi	int	lo
CXCR6	−	−	+	+	+	+	ND	ND	−	−	−
Ly6C	−	ND	ND	−	−	−	ND	ND	lo	int	hi

### cNK Development in the Periphery—NK Cell Maturation

Natural killer cell maturation is a process by which lineage committed NK cells acquire their full effector functions. This process is also accompanied by the expression of different cell surface markers, which have helped in the identification of different NK cell maturation subsets. At present, most studies use CD11b and CD27 to divide cNK cells into three maturation subsets: immature (Imm), mature 1 (M1), and mature 2 (M2). Low (lo) and high (hi) CD11b expression divides cNK cells into immature and mature subsets, respectively (22). Heterogeneity in CD27 expression further delineates the mature NK compartment into CD27^hi^ and CD27^lo^ subsets, which has also been referred to as M1 and M2 NK subsets (24, 28). CD27 and CD11b expressing M1 NK cells have also been termed double-positive NK cells (24). The three subsets differ in proliferative and cytotoxic capacity. In general, NK cells lose proliferative potential and produce less cytokine, but become more cytotoxic against target cells as they mature (22, 24, 28).

Apart from CD27 and CD11b, markers such as KLRG1 (23, 29), CD62L (30), MCAM (31), CD49b (21), CD43 (32, 33), Ly6C (34), DNAM1 (35), and CD160 (36) have further dissected maturing NK cells into various phenotypic subsets (37, 38). NK cells that express CD160 exhibit enhanced IFN-γ production (36), while the opposite is true for mature NK cells that express higher levels of Ly6C (34), and KLRG1 (39). Like Ly6C, MCAM is also more highly expressed on mature NK cells, although MCAM^+^ and MCAM^−^ NK cells differ in their ability to kill target cells rather than cytokine production (31). Interestingly, the expression of DNAM1 appears to be independent of NK cell maturation that is defined by CD27 and CD11b, as DNAM-1^+^ and DNAM-1^−^ NK cells were observed in both the immature and mature NK compartments (35). As the correlation between these markers have not been studied in detail, the relationships between these phenotypic subsets remain unclear and warrants further investigation. A summary of the surface markers that are expressed on the various mature NK cell subsets are provided in Table 1.

## Transcriptional Regulation of Murine cNK Cell Development

Transcription factors control gene expression by either activating or repressing gene transcription. This is achieved by first binding to specific DNA sequences in the enhancer or promoter regions that lay upstream of target genes, then promoting or blocking the recruitment of RNA polymerases that transcribe those genes (40). In terms of murine NK cell development, several TFs have been shown to play crucial roles in regulating NK cell lineage specification, NK cell maturation, or even both. Conventional NK cell development occurs mostly in the bone marrow, under the coordinated control of the TFs and cytokines. TFs like ID2, NFIL3, T-box brain protein 2 (EOMES), and T-box protein 21 (TBET) to name a few, fall into the category of intrinsic factors that regulate NK cell development. A summary of the transcription factors that are implicated in NK cell development is provided in Table 2.

**Table 2:** **Transcription factors implicated in natural killer (NK) cell development and function**.

	Phenotype of germline (KO) or conditional (cKO) deficiency				
Transcription factor (gene symbol)	Bone marrow NK precursor #	NK cells #	IL-15 responsiveness	Interferon-gamma (IFN-γ) production/degranulation	Cytotoxicity (tumor/target cells)
Ikaros family zinc finger 3, Aiolos (*Ikzf3*)	ND	KO: normal; accumulation of immature NKs (iNKs)	KO: hyperresponsive to IL-2/anti-IL-2 mAB complex *in vitro*	KO: slightly impaired IFN-γ production	KO: normal *in vitro* killing, augmented *in vivo* killing

B lymphocyte-induced maturation protein 1 (*Prdm1*)	ND	KO: reduced in spleen, liver, and lung; accumulation in bone marrow and lymph nodes; loss of mNKs	KO: hyperresponsive	KO: normal IFN-γ production	KO: normal *in vitro* killing, augmented *in vivo* killing

T-box brain protein 2, EOMES (*eomesodermin*)	ND	cKO: reduced; loss of mNKs	ND	cKO: slightly impaired IFN-γ production	ND

ETS proto-oncogene 1, ETS1 (*Ets1*)	KO: lack pre-NKPs and rNKPs	KO: reduced	KO: hyperresponsive	ND	KO: impaired *in vitro* killing and degranulation

Forkhead box protein O1, FOXO1 (*Foxo1*)	cKO: normal NKP (41)	cKO: reduced; loss of mNKs (41) cKO: normal; accumulation of mNKs (42)	ND	cKO: augmented IFN-γ production (42)	cKO: augmented *in vitro* and *in vivo* killing (42)

GATA-binding protein 3 (*Gata3*)	ND	cKO: reduced in bone marrow; accumulation in spleen and liver, systemic accumulation of iNKs	ND	cKO: impaired IFN-γ production; normal degranulation	cKO: normal *in vitro* killing

Inhibitor of DNA-binding 2, ID2 (*Id2*)	KO: normal NKP	KO: reduced; loss of mNKs; cKO: systemic reduction	cKO: hyporesponsive	KO: impaired IFN-γ production	KO: impaired *in vitro* killing
				cKO: normal IFN-γ production	cKO: impaired *in vivo* killing

Interferon Regulatory Factor 2 (*Irf2*)	ND	KO: reduced; loss of mNKs	KO: hyporesponsive	KO: impaired IFN-γ production	KO: normal *in vitro* killing (43); impaired *in vitro killing* (44)

Kruppel-like factor 2 (*Klf2*)	ND	cKO: reduced in spleen, blood, and lung; accumulation in bone marow and liver; loss of mNKs	ND	ND	ND

Myeloid elf-1-like factor (*Mef*)	ND	KO: reduced	ND	KO: impaired IFN-γ production	KO: impaired *in vitro* killing

Nuclear factor, interleukin 3 regulated, NFIL3 (*E4bp4*)	KO: lack CILP and NKPs, pre-NKPs and rNKPs	KO: reduced	ND	ND	ND

T-box protein 21, TBET (*Tbx21*)	ND	cKO and KO: reduced in spleen and liver; accumulation in bone marrow; loss of mNKs	ND	KO: impaired IFN-γ production	KO: impaired *in vitro* killing

T-cell-specific transcription factor 1, TCF1 (*Tcf7*)	KO: lack NKPs	KO: reduced in bone marrow; normal in periphery	ND	ND	ND

Thymocyte selection-associated high mobility group box protein (*Tox*)	KO: normal NKP	KO: reduced; loss of mNKs	ND	ND	KO: impaired *in vivo* killing

Zinc finger E-box binding homeobox 2 (*Zeb2*)	ND	KO: reduced in periphery; normal in bone marrow; loss of mNKs	cKO: hyporesponsive	cKO: normal to slightly augmented IFN-γ production	cKO: impaired killing *in vivo*

### Transcription Factors Regulating NK Cell Lineage Specification

The TFs that are involved in regulating NK cell lineage specification include ETS proto-oncogene 1 (ETS1), NFIL3, and TCF1. ETS1 is a key regulator of early NK cell development as ETS1-deficient mice have normal CLP numbers but lack NK cells (45). Further investigation into the impact of ETS1 deficiency on NK lineage specification revealed a reduction in pre-pro NK, pre-NKP and rNKP, and mature NK cell numbers, thereby supporting the role of ETS1 in NK cell lineage specification (46). ETS1 is believed to impact early NK cell development by regulating the expression of ID2 and TBET, which are also important TFs for NK cell development (46).

TCF1 and NFIL3 are also key regulators of NK cell lineage specification, as marked reductions in pre-pro NK, pre-NKP, and rNKP numbers were observed within the bone marrow of NFIL3- and TCF1-deficient mice, although only the former mouse strain exhibited an additional reduction in CILP numbers (25, 26, 47, 48). NFIL3 appears to be dispensable for mature NK cell as their numbers remained unchanged after its deletion in mature NKp46^+^ NK cells (49). Despite reduced progenitor and mature NK cell numbers in the bone marrow of TCF1-deficient mice, mature NK cells have been found in the periphery at frequencies comparable to littermate controls (26, 50). As most NK cells are derived from bone marrow precursors at steady state, an investigation into the source of NK cells in TCF1-deficient mice might perhaps shed some light on alternative pathways of NK development. The mechanism by which TCF1 mediates NK lineage specification remains poorly understood. However, TCF1 has been shown to regulate T-lineage specification by promoting the expression of genes, such as *Gata3, Bcl11b, Il2ra*, and *Cd3e*, that are critical for T cell development (51). Similarly, the mechanism by which NFIL3 specifically mediates lineage specification in NK cells remains unclear, although NFIL3 was found to promote the expression of ID2 and EOMES (47), transcription factors known to be involved in the later stages of NK cell development (52, 53). The role of NFIL3 in ID2 expression remains to be clarified given a contradicting report that ID2 expression is normal in NK cells lacking NFIL3 (48).

The role of ID2 in the lineage specification of NK cells is unclear, due to contradicting reports following deletion of the encoding gene *Id2*. While Yokota et al. (54). reported poor reconstitution of NK cells following bone marrow transplantation, implying a defect early on during NK cell development, Boos et al. (55) did not observe any reduction in NKP and iNK cell numbers. A recent demonstration of low ID2 levels in CLPs but consistently high levels in pre-pro NK, NKP, and immature and mature NK cells lends further support for the hypothesis that ID2 could indeed be important for NK lineage specification/maintenance (53). Interestingly, ID2 has been found to suppress T and B cell development through heterodimer formation with the E-box protein E2A (55–57), although how the interaction specifically promotes commitment to the NK cell lineage remains unknown.

### Transcription Factors Regulating NK Cell Maturation

**Figure 1:**
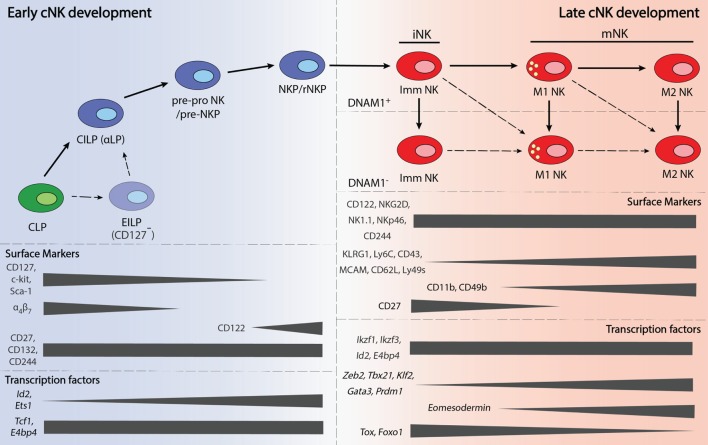
**Overview of the early and late stages of natural killer cell development**.

A greater number of TFs have been shown to play a role in NK cell maturation. These factors include ID2, TBET, EOMES, Zinc finger E-box-binding homeobox 2 (ZEB2), Thymocyte selection-associated high mobility group box (TOX), IKAROS family zinc finger 3 (AIOLOS), Interferon regulatory factor 2 (IRF2), B lymphocyte-induced maturation protein 1 (BLIMP1), Forkhead box O1 (FOXO1), Kruppel-like factor 2 (KLF2), and GATA-binding protein 3 (GATA3). An overview of the expression these TFs during NK cell maturation is presented in Figure 1.

The role of ID2 in NK cell maturation is better understood than its role in early NK cell development. A recent study provided additional insight into the underlying mechanism of ID2 by demonstrating that it modulates the expression of E2A target genes (i.e., *Socs3, Tcf7*, and *Cxcr5*) by titrating E-protein activity, thereby controlling the responsiveness of NK cells to IL-15 that is crucial for survival (53, 58, 59).

TBET and EOMES are members of the T-box family of transcription factors that appear to regulate distinct checkpoints in NK cell maturation. TBET- and EOMES-deficient mice exhibited a similar phenotype where NK cell numbers were reduced in the all lymphoid tissues (52, 60, 61), except in the bone marrow of the former where there was an increase in NK cell numbers (60, 61). Detailed analyses of the bone marrow from TBET-deficient mice revealed that the increase in NK cell numbers was due to an accumulation of iNK cells, which the authors attributed to a developmental block (60). However, the possibility of a defect in NK cell trafficking remains unaddressed, given that TBET plays a role in the expression of sphigosine-1-phosphate receptor 5 (S1P_5_) that is required for NK cell egression from the bone marrow (62). Nonetheless, both TFs are crucial for maturation, as mice that are deficient for both have a systemic lack of NK cells despite normal progenitor numbers (52, 61). As TBET and EOMES are, respectively, required by immature and mature NK cells, the two TFs are believed to function in a sequential manner for NK cell maturation (52, 60). Quite fittingly, both TBET and EOMES were shown to be essential for IL-15 responsiveness by enforcing high CD122 expression, with *Il2rb* (gene encoding CD122) being a direct target of EOMES (63).

Similar to that of TBET-deficient mice, NK cell numbers in the bone marrow of mice deficient for ZEB2 or BLIMP1 were higher than littermate controls (64, 65). The unusual accumulation of NK cells within the bone marrow of ZEB2-deficient mice was due to reduced S1P_5_ expression (64), while enhanced proliferation was found to be the underlying cause in BLIMP1-deficient mice (65). Profound losses in terminally mature NK cells were observed on closer examination of the bone marrow NK cells from both strains (64, 65). The lack of mature NK cells in ZEB2-deficient was further attributed to poor responsiveness to IL-15, which resulted in poor survival (64).

The lack of mature NK cells was reported in mice that were deficient for TOX (66), AIOLOS (67), IRF2 (43, 44), KLF2 (68), or GATA3 (69, 70). A similar lack of mature NK cells in FOXO1-deficient mice was reported most recently (41), although this remains to be clarified against an earlier study, which instead found an accumulation of mature NK cells (42). Interestingly, the iNK cells in AIOLOS-deficient mice retained their expression of KLRG1, which is typically expressed on terminally mature NK cells (67). NK cells from IRF2-deficient mice have been shown to undergo accelerated apoptosis, indicating a role for IRF2 in regulating NK cell survival as well as maturation (43). Unlike the foregoing TFs, the temporal requirement for myeloid elf-1-like factor (MEF) by maturing NK cells has not been determined, as only an overall reduction in NK cell numbers was reported (71).

FOXO1 was recently found to be directly involved in the initiation of autophagy in iNK cells, most likely *via* cytosolic interactions between FOXO1 and the autophagy protein ATG7 (41). KLF2 appears to regulate NK cell maturation *via* a different mechanism, influencing the expression of homing receptors such as CD62L (i.e., L-selectin) on maturing NK cells, thereby dictating their access to IL-15 signaling that is essential for survival (68). Current knowledge of how GATA3 regulates NK cell maturation is limited to disturbances in expression of the TFs ID2, TBET, and NFIL3 (70). Nevertheless, GATA3 has also been shown to regulate NK cell egression from the bone marrow in a CXCR4-dependent manner, and also NK cell proliferation in response to IL-15 *via* CD25 expression (70).

### Transcription Factors Regulating NK Cell Effector Function

Natural killer cell effector function is also regulated by many of the TFs outlined above. FOXO1 has also been proposed to negatively regulate NK cell effector function, as its absence was correlated with augmented IFN-γ production in response to murine cytomegalovirus (MCMV) infections and anti-metastatic activity against the B16F10 mouse melanoma cell line (42). Augmented anti-metastatic activity against the same melanoma cell line also was reported in mice deficient for either BLIMP1 or AIOLOS, despite the lack of any significant impact on cytokine production (65, 67). On the other hand, MEF is required for normal cytokine production and cytotoxicity, as it positively regulates IFN-γ and perforin expression, which corresponds to poorer cytotoxicity against tumor cell targets in MEF-deficient mice (71). Normal cytotoxicity but reduced IFN-γ production has been observed in IRF2-deficient mice and mice specifically lacking GATA3 in NK cells (43, 70). NK cell function is also regulated by TBET and EOMES, as TBET has been shown to bind to the regulatory regions of genes encoding granzyme B and perforin, while the expression of EOMES as NK cells mature is associated with increased transcription of mRNA (52, 60).

## Posttranscriptional Regulation of NK Cell Development by microRNAs (miRs)

microRNAs are short non-coding RNAs (19–26 nt) that modulate gene expression at a posttranscriptional level. Recent studies have shown that miRs are also important for NK cell development and function. Using a Dicer1-deficient mouse model that abrogates miR biogenesis in NK cells, Degouve et al. (72). showed that a 10-fold global reduction in miR expression resulted in reduced NK cell numbers, aberrant NK cell maturation, along with attenuated IFN-γ production and cytotoxicity against target cells. Given that IL-15 signaling *via* the STAT5 and mTOR pathways was significantly affected, it was proposed that miRs regulate NK cell survival by modulating IL-15 sensitivity (72). Although it remains unclear as to whether NK cell survival is dependent on specific miRs, miR-155 and miR-15/16 are unlikely candidates since mice that are deficient for either miR have normal NK cell numbers (73, 74).

Rather than NK cell survival, mIR-155 and miR15/16 appear to be essential for normal NK cell maturation, as NK cells lacking miR-15/16 are unable to fully mature into M2 NK cells (74), much like Dicer1-deficient mice, while miR-155-deficient NK cells undergo accelerated maturation (73). In contrast to the accumulation of M2 NK cells in miR-155 deficient mice, an accumulation of Imm NK cells was observed in mice that over-expressed miR-155, thereby providing additional evidence for the role of miR-155 in NK cell maturation (75). More importantly, the opposing effect that miR-15/16 and miR-155 have on NK cell maturation highlights the pleiotropic effects of miRs and suggests that there is still much to learn about the role of miRs in NK cell biology, particularly about redundancies between miRs. Nevertheless, it has been shown that miR-15/16 controls NK maturation by directly regulating levels of the transcription factor MYB, since the overexpression of miR-15/16 or MYB deficiency in miR-15/16-deficient NK cells rescues the maturation defect (74).

The mechanistic action of miR-155 can be appreciated in the context of NK cell proliferation and homeostasis, as NK cells deficient for miR-155 were unable to proliferate in response to MCMV infections and were also outcompeted by wild-type NK cells when cotransferred into homeostatic or lymphopenic environments (73). This dependency on miR-155 for proliferation under both homeostatic and infectious conditions appears to be mediated through the direct suppression of its target genes suppressor of cytokine signaling 1 (*Socs1*) and pro-apoptotic molecule phorbol-12-myristate-13-acetate-induced protein 1 (*Pmaip1*; encoding NOXA) (73). Interestingly, an accumulation of NK cells was observed in transgenic mice that over-expressed miR-155, lending further support for the regulatory role of miR-155 in NK cell proliferation (75).

## Epigenetic Regulation of NK Cell Development

Histone modifications have previously been shown to be essential events in B and T cell development (76, 77). Recent studies have demonstrated that defects in histone modification also impacts NK cell development with respect to lineage commitment (78) and maturation (79). Mice deficient for enhancer of zeste homolog 2 (EZH2), a H3K27 methyltransferase, were observed to have higher numbers NKPs and NK cells. Microarray analysis revealed that the difference was associated with the upregulation of genes essential for NK cell development and function, thereby resulting in earlier lineage commitment and enhanced survival of NKPs (78). This increase in NK cell production was also observed when hematopoietic progenitors from human and wild-type mice were treated *in vitro* with EZH2 inhibitors (78).

A different type of histone modification, deubiquitination, also appears to be involved in the epigenetic regulation of NK cell maturation. The histone deubiquitinase, MYSM1 (Myb-like, SWIRM, and MPN domains-containing protein 1), was found to regulate NK cell maturation as MYSM1-deficient mice possessed fewer NK cells that were mostly immature (79). Given that no defects were observed in the NKP compartment, MYSM1 was proposed to be uniquely required during NK cell maturation. Mechanistically, MYSM1 regulates NK cell maturation by binding directly to the *Id2* gene locus, as revealed by chromatin immunoprecipitation, thereby maintaining expression of the TF (79). However, the mechanism by which MYSM1 is selectively directed to the *Id2* gene locus remains unclear and thus requires further investigation.

## Conclusion

The discovery of new members within the ILC family has rekindled efforts to better understand the development of NK cells, the founding member of the ILC family. Many of the recent breakthroughs made in the transcriptional regulation of NK cell development have been aided by key tools and techniques such as single cell RNA-seq, *in vitro* differentiation conditions, transcription factor reporter mice and conditional alleles and lineage specific Cre-expressing mouse strains. As these techniques and tools become commonplace in the field of NK cell biology, our understanding of the temporal–spatial transcriptional regulation of NK cell development and the key target genes that govern NK cell fate, homeostasis, and function becomes increasingly more complete. Recent studies have advanced our understanding of how individual TFs may be regulating NK cell commitment and NK cell lineage maintenance. However, how these various TFs form a transcriptional network and act in concert to ensure NK cell homeostasis remains unclear.

On a translational front, defining the extrinsic cues and TFs that regulate NK cell maturation, proliferation, cytokine responsiveness, and priming of effector functions represents an area of therapeutic interest. Proteins that negatively regulate NK cell maturation and fitness are tangible drug targets in cancer immunotherapy as recently evidenced by our group. Building on these potential targets will increase the likelihood of developing specific inhibitors for clinical translation.

## Author Contributions

WG wrote the review. NH conceived and edited the review.

## Conflict of Interest Statement

NH is a cofounder of oNKo-Innate. WG declares no conflict of interest.
